# Chemotherapy resistance in acute myeloid leukemia is associated with decreased anti-tumor immune response through MHC molecule and B7 family members

**DOI:** 10.1007/s12672-024-01072-3

**Published:** 2024-06-11

**Authors:** Jing Ge, Xiaoxuan Yin, Xin Sun, Liduo Kou, Xin Xue, Juan Ma

**Affiliations:** 1 Beijing Shijitan Hospital, Capital Medical University, Beijing, 100038 China; 2 College of Basic Medical Science, Peking University Health Science Center, Beijing, 100191 China; 3 Aerospace Central Hospital, School of Clinical Medicine, Peking University Aerospace, Beijng, 100049 China; 4 China Basic Medical Theory of Chinese Medicine, Academy of Chinese Medical Sciences, Beijing, 100700 China

**Keywords:** AML, Chemotherapeutic drug-resistant, B7 family, MHC molecules

## Abstract

Acute myeloid leukemia (AML) remains challenging due to chemotherapeutic drug-resistance (CDR). Aberrant expression B7 family proteins are involved in tumors evasion. We wonder whether B7 family protein alteration in AML CDR further supports tumor escape. Here, we establish AML cytarabine-resistant cell line U937/Ara-C and report on the expression MHC molecule and B7 family member. HLA-ABC was highly expressed similarly on both cell lines. MIC (MHC class I chain related) A/B and B7-H6 was moderately expressed on the surface of U937 and decreased dramatically by U937/Ara-C. In contrast, enhanced expression of B7-H1 and B7-H7 by U937/Ara-C was observed. HLA-DR and other B7 family members including CD80, CD86, B7-DC, B7-H2, B7-H3, B7-H4, and B7-H5 were not detected by both cell lines. Compared co-cultured with U937, peripheral blood mononuclear cells showed a decreased cytotoxicity when incubated with U937/Ara-C, as indicated by decreased levels of granzyme B and perforin production, accompanied with less TNF-α and lactate dehydrogenase secretion. In conclusion, AML CDR further evades the anti-tumor immune response which may through MHC molecule and B7 family members.

## Introduction

The most common adult acute leukemia, acute myeloid leukemia (AML), due to ungoverned proliferation of malignant clonal hematopoietic cells, is frequently associated with infection, anemia, and bleeding [1]. Currently, cytarabine (Ara-C) is considered as the backbone of chemotherapeutic option throughout AML treatment periods [2, 3]. During virtually all induction regimens, Ara-C is administrated alone or combined with an anthracycline**.** After remission is achieved, Ara-C also serves as the important element for consolidation and maintenance stages [4]. In addition to cytotoxicity of Ara-C in vitro, clinical efficacy to Ara-C therapy depends on the capability of AML blasts accumulation to the active metabolite Ara-C triphosphate, resulting in DNA damage through disturbance of DNA synthesis [5]. Although intensive chemotherapy conducing to initial disease withdrawn, relapse induced by intrinsic or acquired drug resistance signifies a tremendous challenge [6]. In spite of progress in genetic risk stratification and great improvement in therapeutics contributing to amelioration in prognosis for some subgroups, AML is still accompanied with a high mortality [7], with the estimated 5-year overall survival as low as 31.7% [8]. Consequently, the importance of characterizing potential indicators for AML chemotherapeutic drug-resistance has become available, which assists in recognizing patients performing new cancer immunotherapies and target therapies.

Accumulated evidence shows that B7 family members impress both immune responses and cancer development via immunological and non-immunological pathways [9]. The ten B7 family members identified so far are CD80, CD86, B7-H1, B7-DC, B7-H2, B7-H3, B7-H4, B7-H5, B7-H6, and B7-H7. After binding with their specific receptors, they can provide co-stimulatory or co-inhibitory signals, participating in adaptive immune responses and oncogene generation [10, 11]. Aberrant expression B7 family proteins are engaged in cancer progression, affecting the prognosis of AML patients [12–14].

Immune checkpoint molecules, including programmed cell death-1 (PD-1) and programmed cell death ligand-1 (PD-L1), also named B7-H1, play important roles in oncogenesis by maintaining an immunosuppressive tumor microenvironment [15]. The PD-1/PD-L interaction is one of the important approaches for tumors escape from host immune surveillance, and overexpression of B7-H1 is accompanied with poor overall survival rate in AML patients [16]. Moreover, high co-expression of immune checkpoints in blast cells of AML patients correlated with poor outcome FLT3^mut^, RUNX1^mut^, or TET2^mut^ [17]. Overexpression of B7-H6, a ligand for the NK activating receptor NKp30, is correlated with the clinical characteristics of poor prognosis in some AML patients [18]. B7-H7 serves as a potential prognostic indicator, associating with tumor immunity in human pan-cancer, including AML [19]. These molecules play crucial roles in regulating the capability of immune responses, especially T lymphocytes involved, and in tumor evasion [20, 21].

To induce an appropriate immune response, MHC/antigen complexes need to be presented with co-stimulatory molecules and secreting pro-inflammatory cytokines by APC [22]. Tumor cells may evade the immune system by down-regulating the expression of MHC molecules [23]. MIC (MHC class I chain related) A/B is a ligand that activates the NKG2D receptor on NK cells and T cells [24]. However, by lytic shedding MICA/B from cell surface, many human tumors escape this pathway [25]. Shedding reduces immune-stimulatory ligands of NKG2D receptors, also leading to internalization of NKG2D receptors and inhibition of NK cell function [26]. In contrast, impedance of MICA/B shedding promotes anti-tumor responses against AML [27]. Vaccine-induced increase the density of MICA/B proteins on tumor surface enhances the cytotoxic function of NK cell and T cells [28].

Based on these studies, we wonder whether B7 family protein and MHC molecule alteration in AML chemotherapeutic drug-resistant further supports tumor escape. Here, we generated AML Ara-C-resistant cell line, U937/Ara-C, and reported on the expression of MHC molecule and B7 family member. Peripheral blood mononuclear cells exhibited a decreased cytotoxicity when incubated with U937/Ara-C.

## Materials and methods

### Cell preparation

Human AML cell line U937 was purchased from ATCC. The drug-resistant AML cell line U937/Ara-C was generated by exposing of U937 cells stepwise to Cytarabine (Sigma‒Aldrich, St. Louis, MO, USA) of escalating concentrations, ranging from 0.01 μg/ml to 10 μg/ml for one year. In details, cell was suspended in culture medium with low concentration of Ara-C at 0.01 μg/ml as the initial concentration for 24 h. Then Ara-C was removed, and replaced with fresh culture medium. Cell status was observed carefully until it can proliferate normally, and Ara-C concentration was increased to 0.02 μg/ml for another 24 h. The dose of Ara-C was incresed gradually until that cell can survival stablely in medium containg Ara-C at the concentration of 10 μg/ml. To obtain effector cells, healthy blood samples were applied to Ficoll density gradient centrifugation to separate peripheral blood mononuclear cells (PBMCs). All cells were incubated by RPMI 1640 medium with 10% heat-inactivated foetal bovine serum (Ausbian, Australia) at 37 °C and 5% CO_2_.

### Cell growth assay

U937 and U937/Ara-C cells were incubated at the density of 2.5 × 10^5^ cells per well in a 24-well plate. Cells were counted on day 1, day 2 and day3 then the growth curve was drawn and the doubling time was calculated.

### Cell viability assay

U937 and U937/Ara-C cells (1 × 10^5^/well) were cultured alone or containing Cytarabine at the different concentration (0.1 μg/ml, 1 μg/ml and 10 μg/ml) in 96-well flat-bottom plate. Then 1/10 (v/v) Cell Counting Kit-8 (CCK8, Dojindo Laboratories, Kumamoto, Japan) reagent in 100 µl of fresh medium containing was added to each well and incubated for additional time periods as indicated. At 450 nm, the absorbency was measured by a 96-well plate reader (DG5032, Huadong, Nanjing, China).

### Flow cytometry

For cell cycle detection, U937 and U937/Ara-C cells were fixed by precooled 70% ethanol, followed by the addition of 0.5 ml PI/RNase dye. To detect MHC molecules and B7 family proteins on cell surface, PE-conjugated anti-human HLA-ABC, HLA-DR, MIC A/B, CD80, CD86, B7-H1, B7-H2, B7-H4, B7-H5, B7-H6, B7-H7, B7-DC and PE-conjugated isotype controls were purchased from eBioscience (San Diego, CA, USA). PE-conjugated anti-human B7-H3 was provided by R&D System (Minneapolis, MN, USA). The expression of MHC and B7 family protein on U937 and U937/Ara-C cells was tested by staining with its specific antibody for 30 min at 4 °C, then cells were washed twice in phosphate buffered saline (PBS), and suspended in 300 μl PBS. Cells were examined with a flow cytometer, and data were analyzed using the accompanying software (CytExpert, Beckman Coulter, Beijing, China). The histograms were demonstrated for comparation the mean immunofluorescence intensity within the living gated cells between U937 and U937/Ara-C.

### Cytokine ELISA assay

Target cells (U937 or U937/Ara-C) were incubated at 1 × 10^4^/well in a 96-well U-bottom plate, and cocultured with PBMC at an effector-to-target (E/T) ratio of 10:1 for 24 h. Then, the cell-free supernatant was gathered, then secretion of granzyme B (R&D System), perforin (invitrogen) and TNF-ɑ (eBioscience) were measured using its particular human ELISA kit.

### Cytotoxicity assay

Target cells (U937 or U937/Ara-C) were incubated at 1 × 10^4^/well in a 96-well U-bottom plate, co-cultured with PBMC at an E/T ratio of 10:1 for 24 h. The cell-free supernatant was gathered, and cytotoxicity was assessed using a lactate dehydrogenase activity kit (Sigma-Aldrich).

### Statistical analysis

All experiments were performed three times. The data were evaluated by GraphPad Prism 9 software. To assess whether the data were normally distributed, the Shapiro–Wilk normality test was implemented. The results were demonstrated as mean ± SD. Analysis of the differences between two groups was conducted by two-way ANOVA and Student’s t tests. *P* < 0.05 was recognized to be significant statistically.

## Results

### The cell growth characterization of U937 and U937/*Ara*-C

**Fig. 1:**
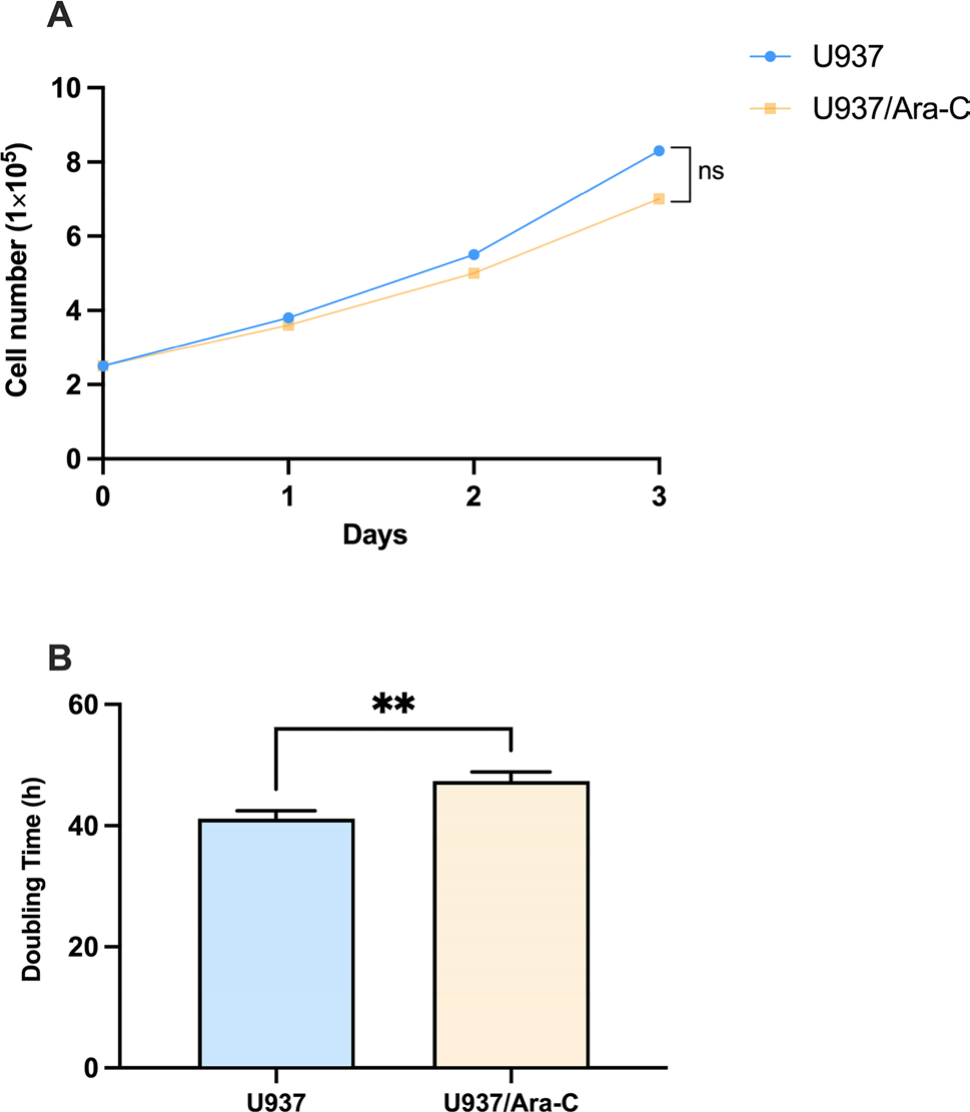
Growth curve and doubling time of U937 and U937/Ara-C. U937 and U937/Ara-C were seeded at the same density, and cells were counted on day 1, day 2, and day 3. Growth curve (**A**) and doubling time (**B**) were determined. A representative result of at least two experiments is shown, and the data are the mean ± SD of triplicate determinations. **P < 0.01, U937/Ara-C was compared with U937 under similar conditions

First, U937, a human AML cell line, and its Cytarabine-resistant, U937/Ara-C, were counted consecutively for three days. As illustrated in Fig. 1, U937/Ara-C grew slower than U937, and the doubling time of U937/Ara-C and U937 were 47.36 ± 1.53 h and 41.17 ± 1.29 h respectively (p < 0.01), demonstrating the growth characterization of drug resistant by U937/Ara-C.

### Resistance of U937/*Ara*-C to Cytarabine

**Fig. 2:**
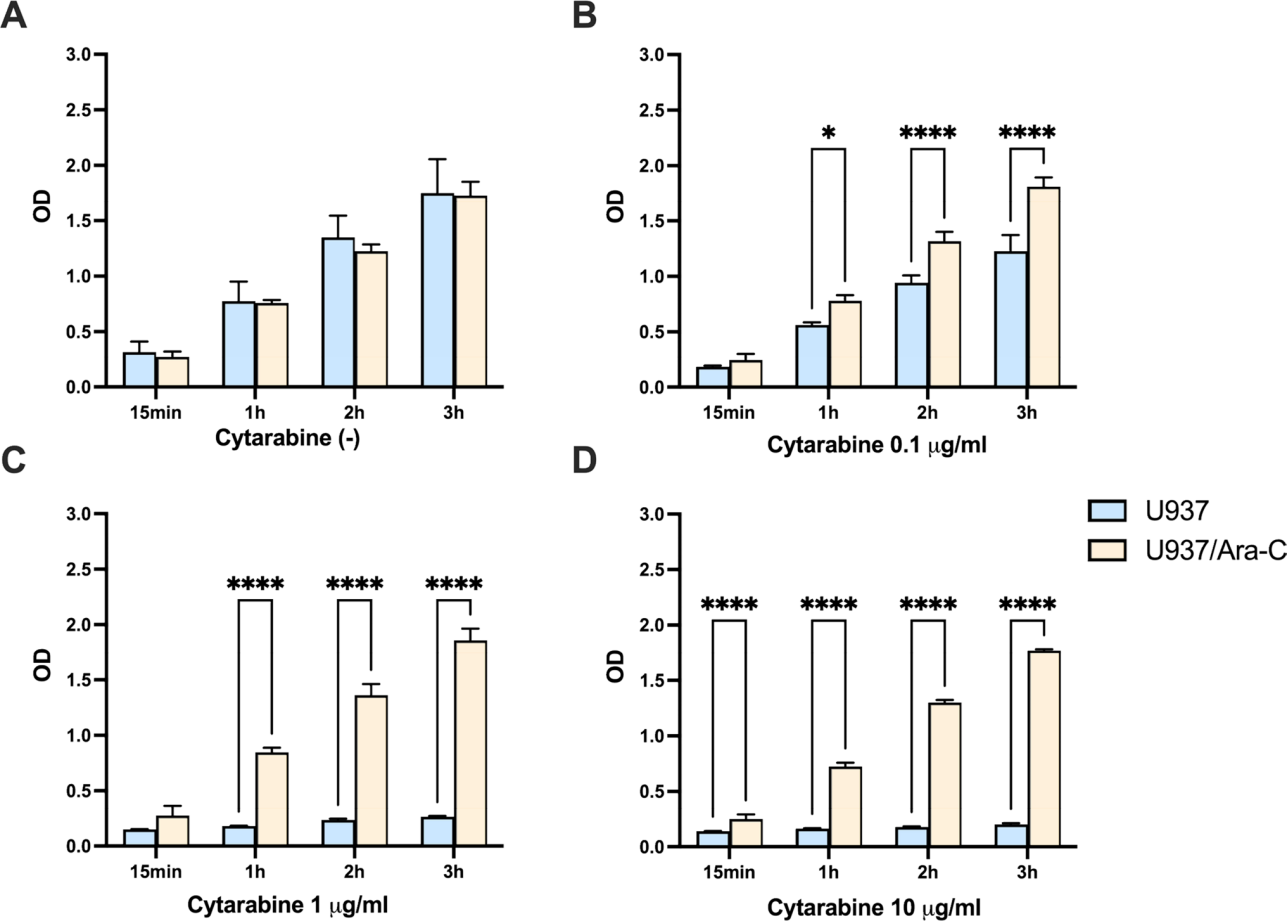
Cytarabine sensitivity of U937 and U937/Ara-C. U937 and U937/Ara-C were cultured without (**A**) or with (**B**-**D**) Cytarabine at the indicated concentration. The absorbency was measured and OD value was detected at different time point. A representative result of at least two experiments is shown, and the data are the mean ± SD of triplicate determinations. *P < 0.05, ****P < 0.0001, U937/Ara-C was compared with U937 under similar conditions

We next analyzed the cell viability of U937 and U937/Ara-C to Cytarabine. As shown in Fig. 2A, U937 and U937/Ara-C proliferated similarly from 15 min to 3 h without Cytarabine. However, in response to Cytarabine treatment, U937 was inhibited significantly with the escalation in drug concentration at 0.1 μg/ml (Fig. 2B), 1 μg/ml (Fig. 2C), and 10 μg/ml (Fig. 2D). On the contrary, the proliferation of U937/Ara-C remained parallel to its control group without Cytarabine, indicating U937/Ara-C but not U937, resistant to Cytarabine from 0.1 μg/ml to 10 μg/ml.

### Reduction in S phase and accumulation in G0/G1 phase by cell cycle of U937/*Ara*-C

**Fig. 3:**
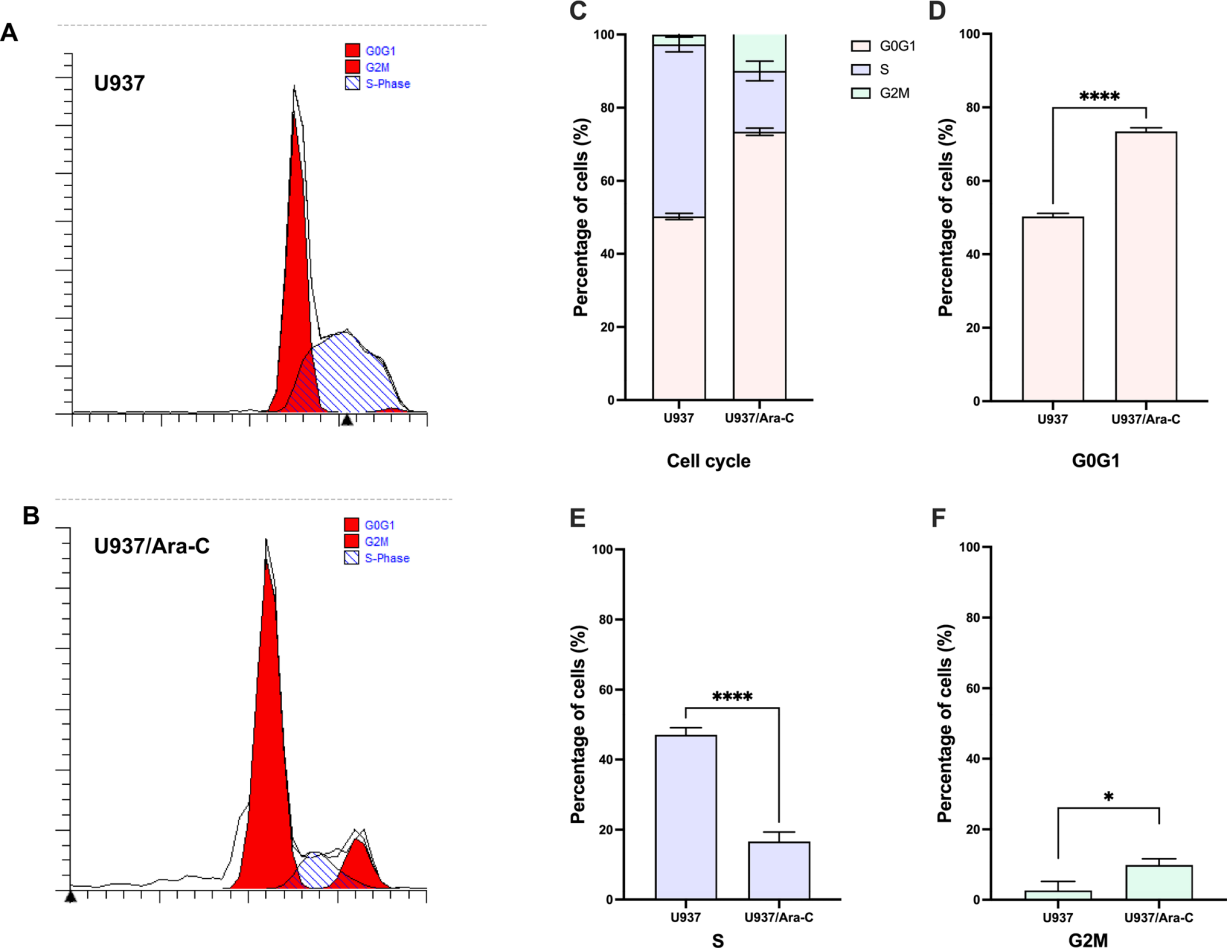
Cell cycle distributions of U937 and U937/Ara-C as detected by flow cytometry. The cell cycle diagrams of U937 (**A**) and U937/Ara-C (**B**). The total population of cell cycle proportion (**C**) in G0/G1 phase (**D**), S phase (**E**), and G2/M phase (**F**) were compared respectively between U937 and U937/Ara-C. A representative result of at least three experiments is shown, and the data for (**C**–**F**) are the mean ± SD of triplicate determinations. *P < 0.05, ****P < 0.0001, U937/Ara-C was compared with U937 under similar conditions

We analyzed cell cycle distribution thereafter. As shown in Fig. 3A, U937 showed a typical G0G1-S-G2M distribution of actively proliferating cells. In comparison, U937/Ara-C demonstrated pronounced decreased proportion in S phase and accretion in G0/G1 and G2/M phases (Fig. 3B), in line with Cytarabine mainly inhibition DNA synthesis in cell cycle. As shown in Fig. 3C, there were significant difference between the U937 and U937/Ara-C in the G0/G1 (Fig. 3D), S (Fig. 3E), and G2/M phase (Fig. 3F) statistically.

### Different pattern of MHC molecule and B7 family protein expression between U937 and U937/*Ara*-C

**Fig. 4:**
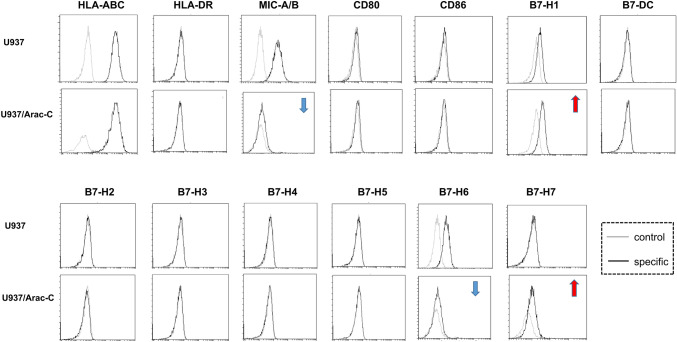
MHC molecule and B7 family member expression on the surface of U937 and U937/Ara-C as detected by flow cytometry. U937 and U937/Ara-C were harvested for immunofluorescence staining and flow cytometry. The Black in histograms denotes fluorescence when stained with the PE-conjugated antibody to the indicated antigen, and the gray line represents fluorescence when stained with the isotype control mAb. Images are representative of at least three experiments

In the following experiment, surface expression of MHC molecules and B7 family proteins was measured via a FACS analysis (Fig. 4). Strong expression of HLA-ABC was observed comparably on both cells lines. On the contrary, HLA-DR expression was not detected on either cell lines. Notably, moderate expression of MIC A/B was observed on the U937, and decreased significantly on the U937/Ara-C. As for B7 family proteins, similarly, moderate expression of B7-H6 was detected by U937 but down-regulated remarkably by U937/Ara-C. Interestingly, enhanced expression of B7-H1 and B7-H7 by U937/Ara-C was observed. Low expression of B7-H1, but not B7-H7 was detected by U937. Other B7 family members including CD80, CD86, B7-DC, B7-H2, B7-H3, B7-H4, and B7-H5 were not detected by both cell lines. These results suggest that compared with U937, U937/Ara-C may further evade anti-tumor immunity through B7-H1, B7-H6, B7-H7 and MIC A/B.

### Decreased cytotoxicity by PBMC against U937/*Ara*-C

**Fig. 5:**
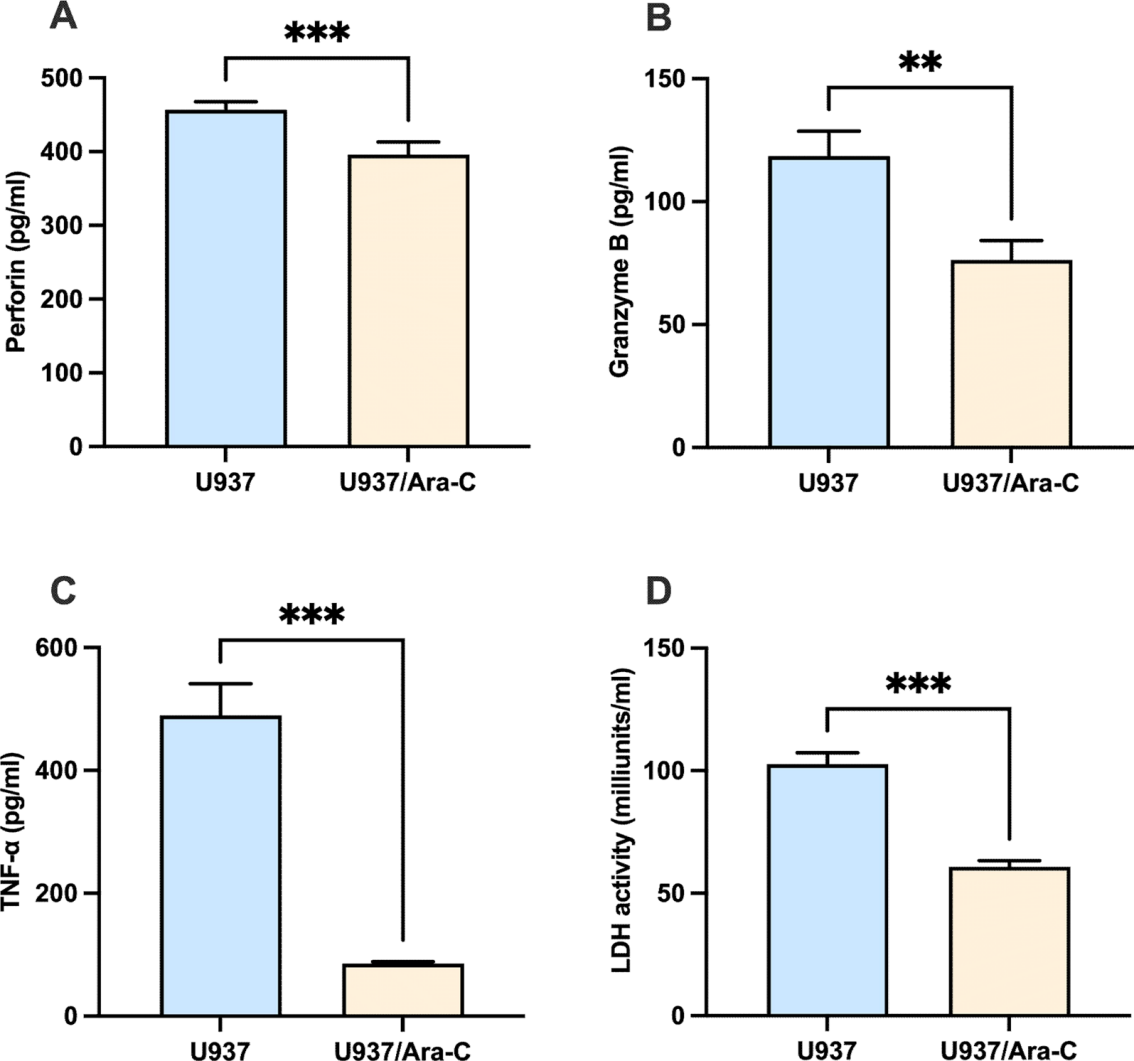
Cytotoxic induction in U937 and U937/Ara-C by PBMC. PBMC were incubated with U937 or U937/Ara-C at an E:T ratio of 10:1 for 24 h, and the supernatant was collected. Then, the levels of secreted perforin (**A**), granzyme B (**B**), TNF-α (**C**), and LDH (**D**) in the supernatant were measured with specific ELISA kits. A representative result of at least three experiments is shown, and the data are the mean ± SD of triplicate determinations. **P < 0.01, ***P < 0.001, PBMC-U937/Ara-C coculture was compared with PBMC-U937 coculture under similar conditions

We next explored peripheral blood mononuclear cells (PBMC) induced cytotoxicity in U937 and U937/Ara-C. PBMC were cocultured with tumour cells at an E:T ratio of 10:1 for 24 h and the coculture supernatant was collected. The amount of the cell-targeted killing-mediating factor released from PBMC was determined. As shown in Fig. 5A and B, the amount of secreted perforin and granzyme B was inhibited after PBMC were cocultured U937**/**Ara-C compared with the amount secreted by PBMC cocultured with U937. The level of secreted TNF-α (Fig. 5C) was also suppressed by PBMC-U937**/**Ara-C coculture compared with PBMC-U937 coculture. Cytotoxicity was also measured by lactate dehydrogenase (LDH) activity assay (Fig. 5D). The level of LDH released by PBMC-U937**/**Ara-C coculture into the culture medium was significantly decreased compared with the amount of LDH released by PBMC-U937 coculture. Taken together, compared with U937, PBMC showed a decreased cytotoxicity to U937/Ara-C.

**Fig. 6:**
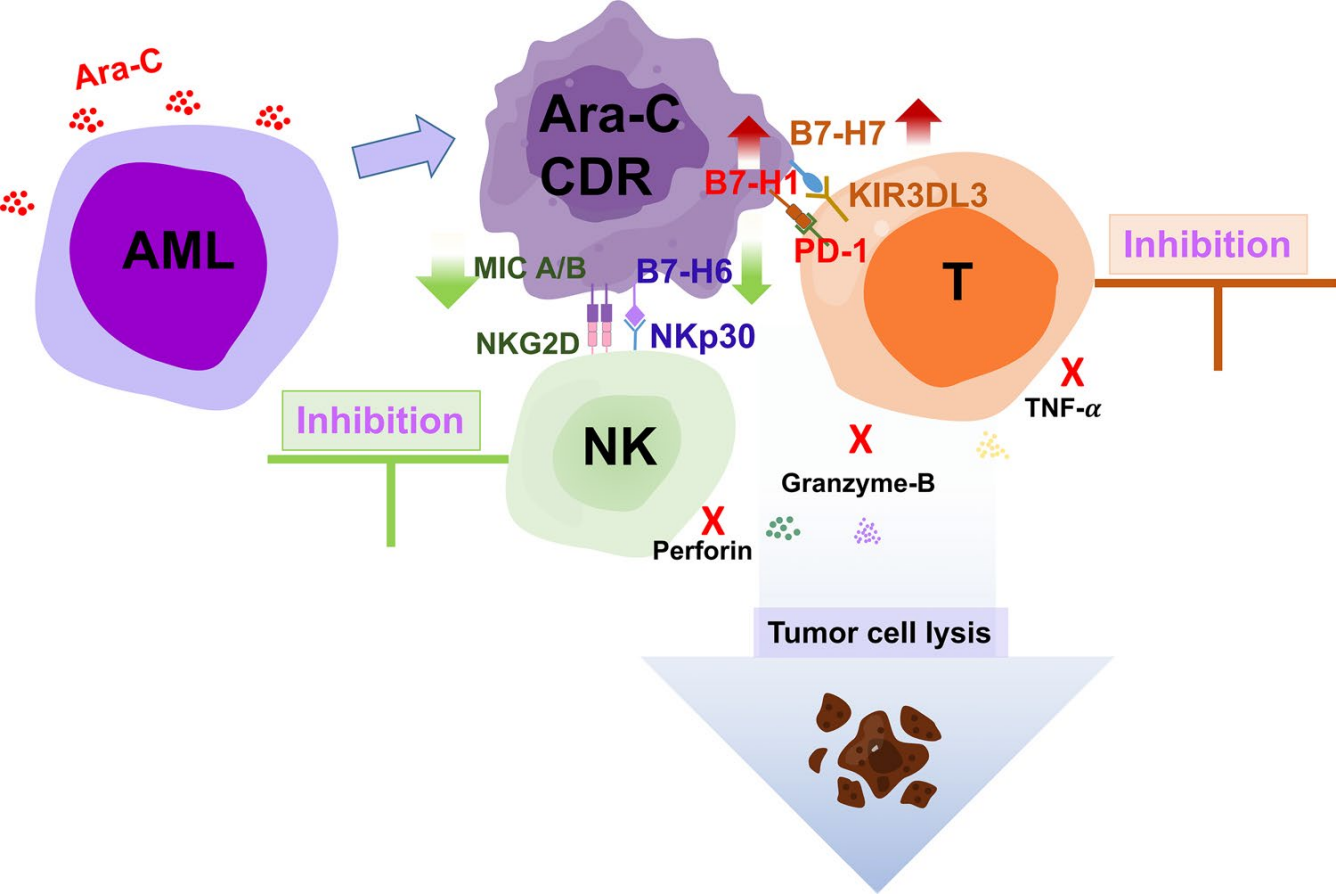
The schematic outline of AML chemotherapeutic drug-resistance further evades the anti-tumor immune response

The scheme AML chemotherapeutic drug-resistance further evades the anti-tumor immune response which may through MHC molecule and B7 family members was summarized in Fig. 6.

## Discussion

Acute myeloid leukemia remains challenging due to chemotherapeutic drug-resistance. Aberrant expression B7 family proteins are involved in tumors evasion. However, whether B7 family protein alteration in chemotherapeutic drug-resistance further supports tumor escape is not determined. Cytarabine is the first-line drug for the treatment of AML. Therefore, in this study, we first generated AML cytarabine-resistant cell line U937/Ara-C. Next, the different expression pattern of MHC molecules and B7 family members was reported on U937 and U937/Ara-C. Then, peripheral blood mononuclear cells were incubated with U937 and U937/Ara-C, respectively. Compared co-cultured with parent U937, peripheral blood mononuclear cells showed a decreased cytotoxicity when incubated with U937/Ara-C, as indicated by decreased levels of killing mediators perforin and granzyme B production, accompanied with less TNF-α and lactate dehydrogenase secretion.

The cytarabine-resistant AML cell line U937/Ara-C was established firstly. Our study showed U937/Ara-C grew slower than U937, consistent with the report that tumors with short doubling times are more sensitive to chemotherapeutic agents [29]. Cytarabine is an anti-metabolite drug that specifically acts on the S phase. It is activated into Ara-C triphosphate in the cell to compete with pyrimidine, inhibiting DNA synthesis and inducing apoptosis [30]. In line with our study U937/Ara-C exhibited reduction in S phase and accumulation in G0G1 and G2M phase by cell cycle. In agreement with its proliferation resistant to cytarabine, altogether, U937/Ara-C was generated successfully resistant to cytarabine in this study.

Recent studies show T-cell senescence and exhaustion, together with impaired NK and T-cell function, are dominant aspects involved in immune dysfunction in AML [31, 32]. Moreover, NK dysfunction was supported by excessive maturation and downregulation of NKG2D and NKP30 [32]. Immune checkpoint therapy’s for targeting CTLA-4 and/or PD-1 overall effectiveness is still lacking, and investigating additional immune checkpoint molecules are popular in recent years [33]. Our study found expression of MIC A/B and B7-H6 decreased dramatically by U937/Ara-C, which may impair NK function through NKG2D and NKP30. On the contrary, we detected expression of B7-H1 and B7-H7 by U937/Ara-C was enhanced, whereas B7-H7 was not detected by U937. The aberrant highly expressed B7 members were correlated with poor prognosis in AML [12], and the PD-1 expression in CD8^+^ T cells was independently predictive of poor overall survival and event-free survival in AML patients [32]. Accordingly, induced expression of B7-H1 and B7-H7 on U937/Ara-C may dampen NK and T-cell function [34–36]. Moreover, B7-H1 on tumors also interacts with CD80 on T cell, inhibiting T cell responses in vivo [37].

After recognition target cells, cytotoxic T lymphocyte (CTL) secrete soluble granzyme–perforin complexes, and perforin forms pores on the cell membrane to help granzyme entering the target cells, which are thus lysed [38, 39]. TNF-α, the important cytokine responsible for the body's immune defense, induces vascular function damage and disrupts tumor blood supply, leading to tumor necrosis [40]. Cytotoxicity was also measured by lactate dehydrogenase activity assay, for that LDH reflects the degree of cell damage and necrosis. Therefore, decreased levels of killing mediators perforin and granzyme B production, accompanied with less TNF-α and lactate dehydrogenase secretion, suggests impaired cytotoxicity by PBMC in our study when co-cultured with U937/Ara-C. Further study by combining the analysis of primary AML patients’ samples and in vivo AML animal models to test separately the different mononuclear fraction component will better clarify the mechanism involved in B7 mediated killing.

As we konown, immune responses are tightly regulated by immune checkpoints including B7 family members to ensure both optimal activation and protective immunity and tolerance. However, tumors exploit these pathways to evade eradication by the immune system. The mechanistic understanding has provided a basis for developing targted immunotherapies for AML with abnormal expression of these checkpoints, and specific immunotherapies are required to target the distinct immune evasion strategies for AML. In summary, our results indicate that AML chemotherapeutic drug-resistance further evades the anti-tumor immune response which may through MHC molecule and B7 family members. Although focusing on the U937/Ara-C at the cellular level, due to AML backbone therapy with cytarabine, our findings suggest combination strategies for overcoming drug resistance and improving the prognosis of patients.

## Data Availability

The data that support the findings of this study are available from the corresponding author upon reasonable request.
